# Diagnosis and treatment of juvenile yellow granuloma in a child: A case report

**DOI:** 10.1097/MD.0000000000046357

**Published:** 2026-01-16

**Authors:** Jian Zhu, Qiang Li, Yifei Dong

**Affiliations:** a Department of Orthopedics, The Second Affiliated Hospital of Zhejiang University School of Medicine, Hangzhou, Zhejiang, PR China; b Department of Pediatric Orthopedics, Beijing Jishuitan Hospital, Beijing, PR China.

**Keywords:** epiphyseal plate preservation, imaging nonspecificity, immunohistochemistry, juvenile xanthogranuloma

## Abstract

**Introduction:**

Juvenile yellow granuloma (JXG) is a reactive proliferation of histiocytes rather than a true neoplasm. We present an extremely rare case of osseous JXG, which highlights the nonspecific imaging features and diagnostic challenges associated with this lesion.

**Case presentation:**

This report describes the diagnosis and management of JXG in the proximal tibia of a 4-year-old Asian girl. The lesion was incidentally discovered following an ankle sprain. Imaging studies (X-ray, CT, MRI) initially suggested eosinophilic granuloma. Laboratory tests revealed elevated high-sensitivity C-reactive protein (43.84 mg/L) and erythrocyte sedimentation rate (63 mm/h). Biopsy revealed giant cell-rich tumors with foam cell aggregates. Immunohistochemistry was consistent with xanthoma. Given the large lesion size (6.8 × 3.5 × 3.1 cm) and involvement of the epiphyseal plate, the patient underwent robot-assisted curettage, implantation of artificial bone/cement, Kirschner wire fixation, and cast immobilization. Final pathological diagnosis, confirmed by immunohistochemistry (CD68+, CD163+, S-100−), was JXG.

**Conclusions:**

This case underscores the rarity and imaging nonspecificity of osseous JXG, emphasizing the critical role of histopathology combined with immunohistochemistry for definitive diagnosis. A multimodal treatment approach involving curettage, epiphyseal plate protection, and mechanical stabilization achieved a successful outcome. Long-term follow-up is necessary to monitor for potential epiphyseal injury and recurrence.

## 1. Introduction

Juvenile yellow granuloma (JXG) is not a true tumor but a reactive proliferation of histiocytes, thought to originate from a subpopulation of non-Langerhans dendritic cells known as dermal dendritic cells. The case presented here is exceptionally rare and illustrates the nonspecific imaging manifestations of this lesion and the diagnostic difficulties encountered even with pathological examination.

## 2. Case presentation

A 4-year-and-3-month old Asian girl presented with a lesion in the proximal right tibia discovered on October 15, 2024.

### 2.1. History

The lesion was incidentally identified on radiographs taken at a local hospital following a sprain. The patient was referred to our institution for further management. Outpatient CT scans raised suspicion for eosinophilic granuloma, leading to hospital admission. Her medical history was unremarkable.

### 2.2. Physical examination

Examination revealed medial swelling of the right knee without subcutaneous ecchymosis, varicose veins, or focal tenderness. Muscle strength in the bilateral quadriceps and biceps was grade 5. The range of motion for knee flexion and extension was −10° to 135° bilaterally. Both lower limbs were equal in length without deformity, knee rotation abnormalities, or neurovascular deficits.

### 2.3. Laboratory tests

Results were as follows: hemoglobin 114 g/L, platelet count 425 × 10^9^/L, white blood cell count 8.64 × 10^9^/L, absolute eosinophil count 0.12 × 10^9^/L (normal: 0.00–0.68 × 10^9^/L), high-sensitivity C-reactive protein 43.84 mg/L (normal: 0–3 mg/L), erythrocyte sedimentation rate 63 mm/h (normal: 0–15 mm/h), alkaline phosphatase 251 IU/L (normal: 143–406 IU/L), total cholesterol 5.13 mmol/L (normal: <5.18 mmol/L), triglycerides 1.24 mmol/L (normal: <1.7 mmol/L), HDL cholesterol 1.66 mmol/L (normal: 1.29–1.55 mmol/L).

## 3. Imaging studies

**Figure 1.**
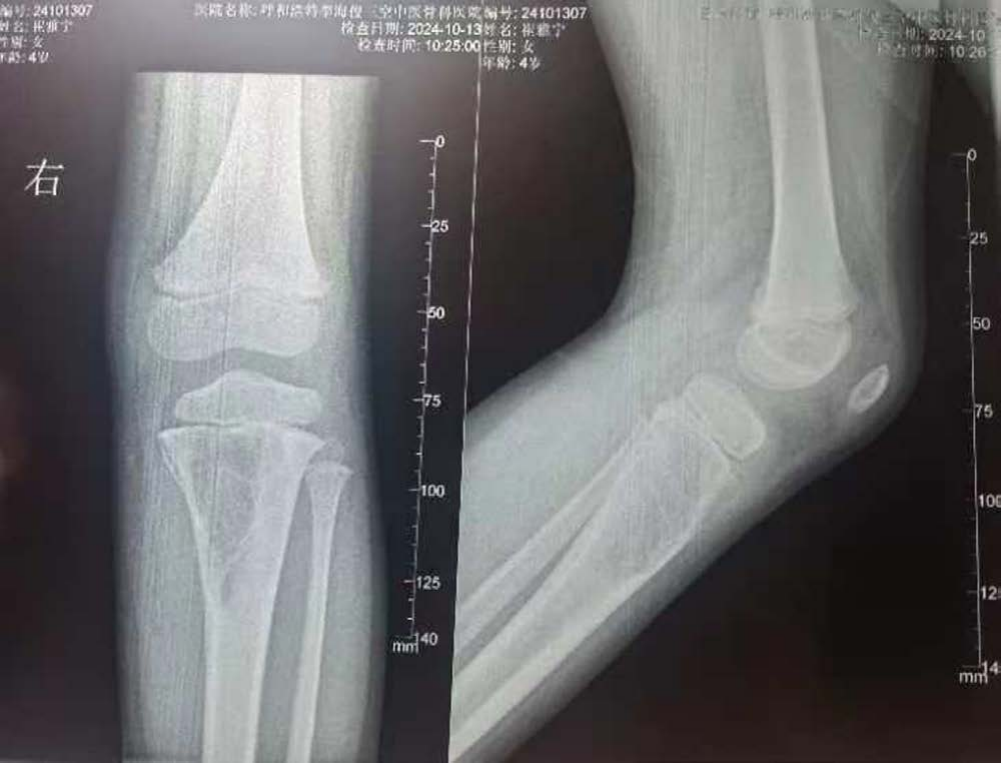
Bone destruction of the proximal tibia on the right side with localized bone mineral density reduction.

**Figure 2.**
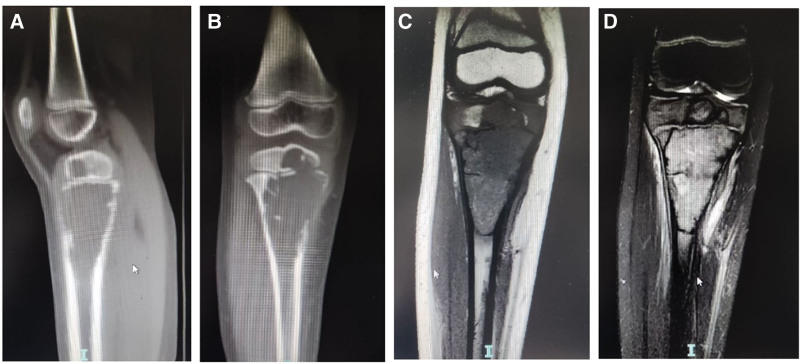
CT sagittal plane (A) and coronal plane (B) showed distended osteolytic bone destruction of the right proximal tibia metaphysis and bone, with mild sclerosis of part of the margins, bone crests on the edges, localized interruption of the bone cortex, and swelling of the surrounding soft tissues. t1 image (C) and t2 image of MRI showed bone destruction foci of the right proximal tibia, which was slightly distended with a clear boundary, sclerosis of part of the margins, involvement of the metaphyseal end and epiphysis, and localized disruption of the adjacent bone cortex. The lesion is about 6.8*3.5*3.1 cm (upper and lower + left and right + anterior and posterior), with T1WI equal and T2WI high signal, accompanied by multiple bone crests. Diffuse edema of soft tissues around the lesion. The right fibula did not show clear signs of bone destruction.

**Figure 3.**
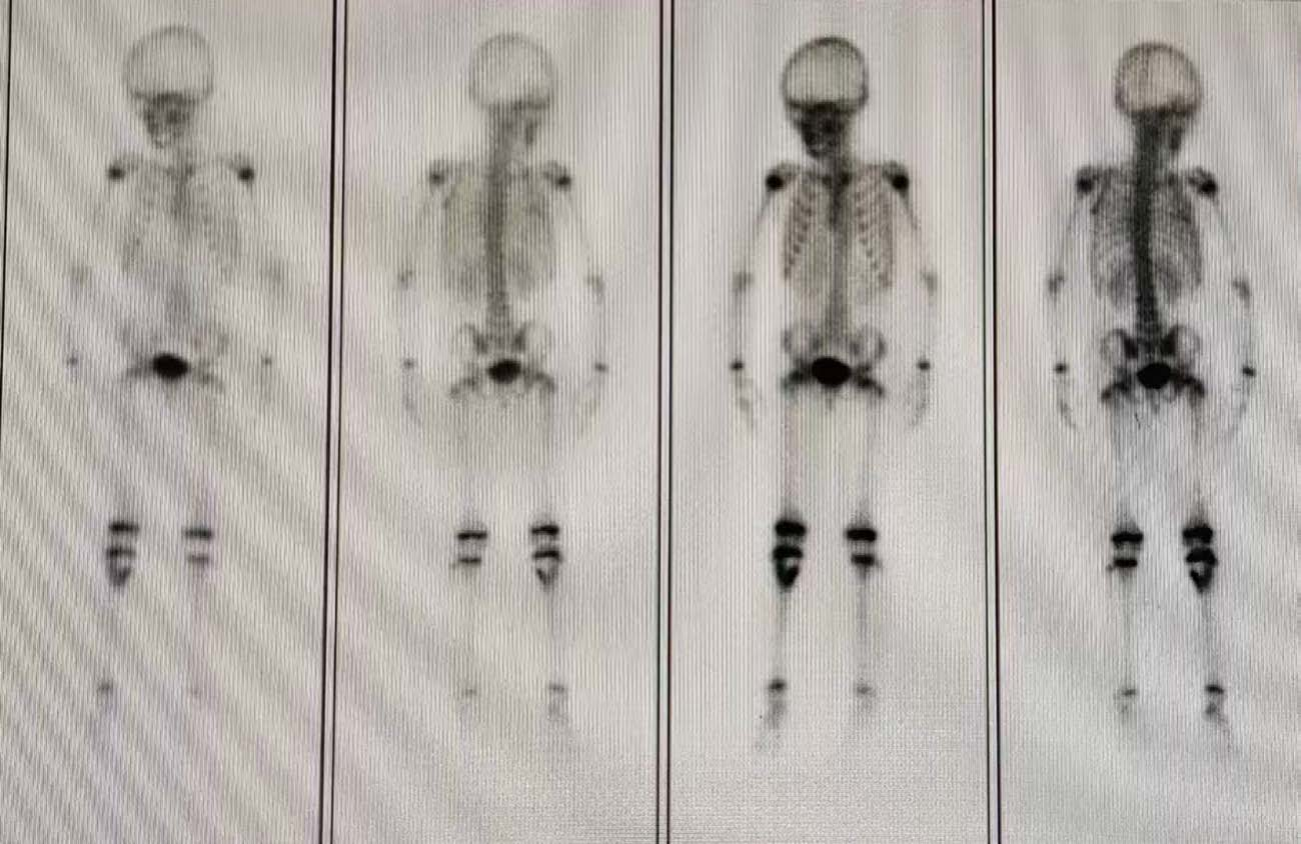
Right proximal tibia lesion with radiologically distributed inhomogeneous hyperintensities in the right proximal tibia, no abnormal lesions were seen in other parts of the skeleton.

- **Ultrasound:** Showed bone destruction in the proximal right tibia with an internal solid hypoechoic mass and slightly abundant blood flow, suggestive of eosinophilic granuloma.
- **X-ray:** Confirmed bone destruction in the proximal right tibia (Fig. 1).
- **CT:** Revealed an osteolytic lesion in the proximal right tibia, possibly an eosinophilic granuloma (Fig. 2A and B).
- **MRI:** Demonstrated a slightly expansile, well-defined destructive lesion in the proximal right tibia measuring approximately 6.8 × 3.5 × 3.1 cm. The lesion involved the metaphysis and epiphysis, with localized cortical disruption. It showed iso-intensity on T1-weighted images and high signal intensity on T2-weighted images, containing multiple septa. Surrounding soft tissue edema was noted (Fig. 2C and D). Eosinophilic granuloma was considered.
- **Whole-body bone scintigraphy:** Showed inhomogeneously increased radiotracer uptake in the proximal right tibia, with no other skeletal abnormalities detected (Fig. 3).

A biopsy of the right proximal tibia lesion was performed on October 25, 2024. The initial immunohistochemical results (Wax block: X2404656-A4) were: S-100 (scattered +), CD163 (+), CK (AE1/AE3) (−), CD68 (+), H3.3K36M (−), H3.3G34W (−), H3.3G34R (−).

**Figure 4.**
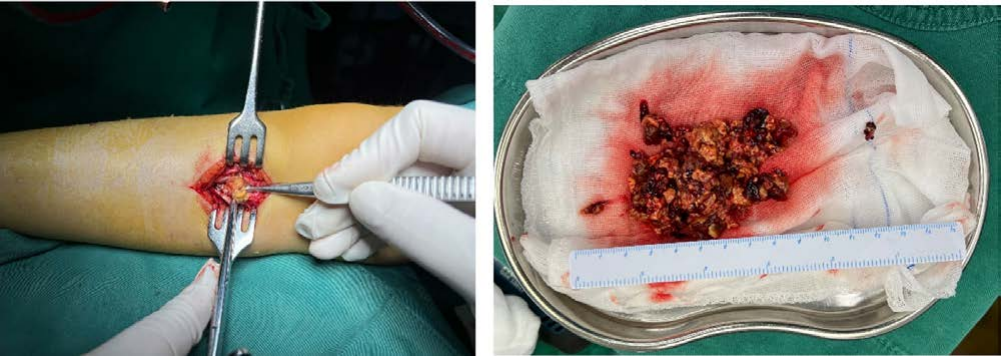
Intraoperative thorough scraping of the lesion with a scraping spoon, a large amount of yellow tofu-dregs-like tissue was scraped out, which was soft in texture.

On November 29, 2024, after excluding surgical contraindications, the patient underwent robot-assisted tumor resection in the right proximal tibia. Intraoperatively, the lesion was thoroughly curetted, yielding a large amount of soft, yellow, tofu-dregs-like tissue (Fig. 4).

### 3.1. Postoperative pathology

Microscopic examination showed proliferating histiocytes mixed with a small number of chronic inflammatory cells, scattered multinucleated giant cells, occasional Touton-like giant cells, patchy necrosis, focal cholesterol clefts, and aggregates of foam cells. The initial pathological impression was insufficient to support giant cell tumor or other histiocytic tumors, and JXG was primarily considered based on the overall clinicopathological picture, a conclusion reached after departmental consultation. The final immunohistochemistry results (Wax block: X2405345-A1) were H3.3K36M (−), H3.3G34W (−), H3.3G34V (−), H3.3G34R (−), CD207 (−), S-100 (−), P63 (−), Vimentin (+), CK (AE1/AE3) (−), CD68 (+), CD31 (+), CD163 (+), and ALK (5A4) (−).

## 4. Discussion and conclusion

In this case, a 4-year-old Asian child with no significant pain was found to have a proximal tibial lesion on imaging after a minor trauma. Initial X-ray revealed bone destruction. Subsequent ultrasound, CT, and MRI findings were all suggestive of eosinophilic granuloma. Since eosinophilic granuloma can present as single or multiple lesions, a bone scan was performed, which confirmed the lesion was solitary. Eosinophilic granuloma typically presents as a well-defined osteolytic lesion, potentially with bone expansion, periosteal reaction, or peripheral sclerosis, often located in the medullary cavity. However, given the nonspecific imaging features – such as bone destruction involving the metaphysis and endosteum, along with cortical discontinuity – it is crucial to differentiate it from osteomyelitis, primary bone malignancies, metastatic tumors, and common benign bone tumors. Patients with osteomyelitis often exhibit leukocytosis, a prior infection history, and local signs of inflammation. Primary bone malignancies frequently show periosteal reactions, while metastatic lesions have known primary sites. Common benign tumors like unicameral bone cysts are well-defined without sclerotic borders; aneurysmal bone cysts are typically expansile and eccentric with fluid-fluid levels on MRI; giant cell tumors of bone are eccentric and osteolytic with a “soap-bubble” appearance; and fibrous dysplasia often has a “ground-glass” or “worm-eaten” appearance. Based on the history, physical exam, and initial investigations, eosinophilic granuloma, a manifestation of Langerhans cell histiocytosis (LCH) which accounts for 60 to 80% of LCH cases,^[1]^ was considered highly likely.

Xanthoma, a benign lesion associated with abnormal lipid metabolism, results from cholesterol and lipid deposition leading to inflammatory cell infiltration.^[2,3]^ Radiographs of intraosseous xanthoma typically show a well-marginated lytic lesion, often with sclerotic borders.^[4]^ CT scans reveal osteolysis, sometimes with cortical destruction.^[5]^ MRI typically demonstrates a well-defined lesion with heterogeneous signal on both T1- and T2-weighted images, showing signal loss on fat-suppressed sequences due to cholesterol-laden histiocytes.^[6,7]^ SPECT may show a ring of increased uptake.^[8]^ The imaging findings in this case were consistent with these features. The histopathological hallmark of intraosseous xanthoma is the presence of lipid-rich histiocytes (foam cells) with abundant cytoplasm and small nuclei, surrounded by fibrous tissue with minimal inflammation.^[9,10]^ Immunohistochemically, these lesions are strongly positive for CD68, a marker for macrophages.^[11]^ Curettage is an effective treatment that promotes bone regeneration,^[11]^ and prophylactic fixation may be necessary for large osteolytic lesions to prevent pathological fractures.^[12]^

Consequently, we performed curettage of the lesion followed by bone grafting to facilitate healing. Filler was placed at the epiphyseal level to prevent bone bridge formation. The limb was supported with Kirschner wire fixation to prevent fracture deformity, supplemented by a cast. The final pathology, showing Touton-like giant cells, cholesterol deposition, and foam cells, with immunohistochemistry ruling out other histiocytic tumors, confirmed the diagnosis of JXG.

JXG is a reactive histiocytic proliferation, potentially arising from dermal dendritic cells, a subset of non-Langerhans dendritic cells.^[13]^ It typically affects infants and children. Its etiology remains unknown. JXG commonly presents as soft, yellow papules or nodules on the head and neck. While over 90% of cases are confined to the skin, visceral involvement (e.g., liver, spleen, lungs, eyes, bone, CNS) can occur. Systemic symptoms with skeletal involvement have been reported, but isolated bone lesions are rare.^[14]^

Histologically, JXG is characterized by histiocytic proliferation with foamy xanthoma cells, Touton giant cells, and a mixed inflammatory infiltrate including lymphocytes and eosinophils.^[15]^ The diagnostic immunophenotype is positive for Factor XIIIa, CD68, CD163, and CD14, but negative for CD1a and S-100.^[16,17]^

The histological differential diagnosis includes other macrophage/histiocyte-derived disorders such as LCH, unicameral bone cyst, Erdheim-Chester disease, and Rosai-Dorfman disease.^[18–21]^ LCH involves clonal proliferation of Langerhans cells with characteristic coffee-bean nuclei and immunopositivity for CD1a, CD207 (Langerin), and S-100, while being negative for Factor XIIIa. Erdheim-Chester disease is a rare systemic non-LCH featuring xanthogranulomatous infiltrates, Touton giant cells, and fibrosis in multiple organs.^[22]^ Rosai-Dorfman disease is a polyclonal histiocytic disorder. In contrast, xanthomas are primarily composed of foamy histiocytes and multinucleated giant cells, often with extracellular cholesterol deposits.^[23,24]^

Cutaneous JXG is often self-limiting and may regress spontaneously.^[19]^ However, spontaneous resolution has not been reported for bone lesions. Complete excision typically leads to favorable outcomes without recurrence. Adjuvant chemotherapy or radiotherapy has been used for partially resected cases.^[20,25,26]^ Cao et al recommend close follow-up for partially resected patients, with reoperation if symptoms recur.^[27,28]^ Only one previous case of JXG involving the tibia has been reported worldwide, which was treated with curettage, bone grafting, and internal fixation.^[24]^

This case is exceptionally rare. The diagnostic journey – from imaging suggestive of eosinophilic granuloma, to biopsy hinting at xanthoma, and finally to postoperative pathology confirming JXG – demonstrates the nonspecific imaging presentation and pathological diagnostic challenges. The general principles for managing solitary benign bone lesions include observation, biopsy for diagnosis, curettage and grafting to eradicate the lesion, internal fixation to prevent fracture, and specific measures to address epiphyseal plate involvement to prevent growth disturbances. In this case, the large lesion size and epiphyseal involvement warranted surgical intervention. The treatment involved curettage, distilled water irrigation, epiphyseal plate protection with bone cement, artificial bone grafting, and Kirschner wire fixation. However, since damage to the epiphyseal plate had already occurred, long-term follow-up is essential to monitor for potential recurrence, bone bridge formation, and growth-related complications.

This case offers several critical insights for clinicians managing osteolytic bone lesions in children. From our perspective, the most significant lesson is the profound limitation of imaging in providing a definitive diagnosis for rare entities like JXG. Our initial working diagnosis, strongly supported by multiple imaging modalities, was eosinophilic granuloma. This was a logical assumption given the patient’s age, the lesion’s location, and its radiographic features. This experience underscores that JXG can be a perfect “mimicker” of more common bone pathologies.

The diagnostic challenge extended to the pathological examination. The initial biopsy was inconclusive, highlighting that small tissue samples may not capture the full histopathological spectrum of JXG, particularly characteristic features like Touton giant cells. It was only upon examination of the entire curetted specimen that the diagnosis became clear. This leads us to a key personal recommendation: when dealing with a destructive bone lesion with atypical or nonspecific features on biopsy, a multidisciplinary consultation involving radiologists, oncologists, and pathologists is essential. In our case, the departmental pathology consultation was a pivotal step towards the correct diagnosis.

The management of solitary bone JXG is primarily surgical, as spontaneous regression – common in cutaneous forms – has not been reported for intraosseous lesions. Our surgical strategy was guided by 2 main concerns: eradicating the lesion and preserving future growth. The lesion’s large size and involvement of the epiphysis and growth plate mandated a proactive approach. We believe that the combination of thorough curettage, distilled water irrigation, and the use of bone cement as a physical barrier at the epiphyseal plate was crucial to minimize the risk of recurrence and growth arrest. The addition of Kirschner wire fixation provided immediate stability to prevent pathological fracture in a weight-bearing bone. While this approach appears effective, the long-term consequences of growth plate damage are unknown. Therefore, our most important conclusion is that this patient requires stringent, long-term follow-up until skeletal maturity to monitor for recurrence, limb length discrepancy, or angular deformity.

In conclusion, intraosseous JXG is exceptionally rare but should be considered in the differential diagnosis of osteolytic lesions in children, especially when the clinical picture is not entirely typical for LCH. Its imaging features are nonspecific, and definitive diagnosis relies on a thorough histopathological and immunohistochemical examination, often requiring expert consultation. Complete curettage is the treatment of choice. For lesions involving the epiphysis, a meticulous surgical technique aimed at protecting the growth plate is recommended, followed by lifelong monitoring for potential skeletal complications. This case serves as a reminder that in pediatric orthopedics, rare diagnoses, though uncommon, must remain on the radar to ensure accurate diagnosis and appropriate management.

## Author contributions

**Data curation:** Qiang Li.

**Funding acquisition:** Jian Zhu.

**Investigation:** Qiang Li.

**Supervision:** Yifei Dong.

**Writing – original draft:** Jian Zhu.

## References

[1] Angelini A Mavrogenis AF Rimondi E Rossi G Ruggieri P. Current concepts for the diagnosis and management of eosinophilic granuloma of bone. J Orthop Traumatol. 2017;18:83–90. 27770337 10.1007/s10195-016-0434-7 PMC5429252

[2] Han Y Gao W Liang P Wang S Chen Y Qiu J. Clinical features of bilateral temporal bone xanthoma with LDLR gene mutation. Int J Pediatr Otorhinolaryngol. 2015;79:1148–51. 25921077 10.1016/j.ijporl.2015.04.020

[3] Blobstein SH Caldwell D Carter M. Bone lesions in xanthoma disseminatum. Arch Dermatol. 1985;121:1313–7. 4037827

[4] Bertoni F Unni KK McLeod RA Sim FH. Xanthoma of bone. Am J Clin Pathol. 1988;90:377–84. 3140652 10.1093/ajcp/90.4.377

[5] Wang Z Lin ZW Huang LL. Primary non-hyperlipidemia xanthoma of bone: a case report with review of the literature. Int J Clin Exp Med. 2014;7:4503–8. 25558299 PMC4280051

[6] Shin WC Moon NH Suh KT. Primary intraosseus xanthoma involving the proximal femur in a normolipidemic patient: a case report. Hip Pelvis. 2016;28:182–6. 27777923 10.5371/hp.2016.28.3.182 PMC5067397

[7] Yamamoto T Kawamoto T Marui T. Multimodality imaging features of primary xanthoma of the calcaneus. Skeletal Radiol. 2003;32:367–70. 12719924 10.1007/s00256-003-0627-z

[8] Lojo-Ramirez JA Garcia-Gomez FJ Kaen A Roldan F Marcilla-Plaza D Acevedo-Banez I. (99m)Tc-HDP SPECT/MRI in isolated xanthoma of the temporal bone. Rev Esp Med Nucl Imagen Mol. 2015;34:329–30. 26198794 10.1016/j.remn.2015.01.002

[9] Daley T Dunn G Darling MR. Central xanthoma of the jaws: a clinicopathologic entity? Oral Surg Oral Med Oral Pathol Oral Radiol. 2015;119:92–100. 25446505 10.1016/j.oooo.2014.09.018

[10] Wilkinson PE Merkourea S Gopalakrishnan R Argyris PP. Primary intraosseous xanthomas of the jaws: a series of six cases including an example with formation of apoptosis-related hyaline globules, so-called “Thanatosomes”. Head Neck Pathol. 2020;14:859–68. 31916206 10.1007/s12105-020-01126-2 PMC7669974

[11] de Arruda JAA Almeida TFA Abreu LG. Intraosseous xanthoma of the mandible: a multi-institutional case series with a literature review. J Oral Pathol Med. 2019;48:935–42. 31355943 10.1111/jop.12940

[12] Schryver EM McCandless MG Al Hmada Y Barr JS. Multifocal xanthoma of bone. J Am Acad Orthop Surg Glob Res Rev. 2021;5:e20.00261–4. 34010236 10.5435/JAAOSGlobal-D-20-00261

[13] De Paula AM Andre N Fernandez C. Solitary, extracutaneous, skull-based juvenile xanthogranuloma. Pediatr Blood Cancer. 2010;55:380–2. 20582967 10.1002/pbc.22534

[14] Wang SN Lu J. Solitary juvenile xanthogranuloma of temporal bone: a case report. BMC Pediatr. 2022;22:87. 35151291 10.1186/s12887-022-03150-3 PMC8840227

[15] Farrugia EJ Stephen AP Raza SA. Juvenile xanthogranuloma of temporal bone--a case report. J Laryngol Otol. 1997;111:63–5. 9292136 10.1017/s002221510013645x

[16] Weitzman S Jaffe R. Uncommon histiocytic disorders: the non-Langerhans cell histiocytoses. Pediatr Blood Cancer. 2005;45:256–64. 15547923 10.1002/pbc.20246

[17] Cornips EM Cox KE Creytens DH Granzen B Weber JW Laak-Poort MPT. Solitary juvenile xanthogranuloma of the temporal muscle and bone penetrating the dura mater in a 2-month-old boy. J Neurosurg Pediatr. 2009;4:588–91. 19951050 10.3171/2009.7.PEDS09230

[18] Orvets ND Mayerson JL Wakely PE Jr. Extranodal rosai-dorfman disease as solitary lesion of the tibia in a 56-year-old woman. Am J Orthop (Belle Mead NJ). 2013;42:420–2. 24078967

[19] Janssen D Harms D. Juvenile xanthogranuloma in childhood and adolescence: a clinicopathologic study of 129 patients from the kiel pediatric tumor registry. Am J Surg Pathol. 2005;29:21–8. 15613853 10.1097/01.pas.0000147395.01229.06

[20] Dehner LP. Juvenile xanthogranulomas in the first two decades of life: a clinicopathologic study of 174 cases with cutaneous and extracutaneous manifestations. Am J Surg Pathol. 2003;27:579–93. 12717244 10.1097/00000478-200305000-00003

[21] Konar S Pandey P Yasha TC. Solitary juvenile xanthogranuloma in cervical spine: case report and review of the literature. Turk Neurosurg. 2014;24:102–7. 24535803 10.5137/1019-5149.JTN.7712-13.0

[22] Abdelfattah AM Arnaout K Tabbara IA. Erdheim-chester disease: a comprehensive review. Anticancer Res. 2014;34:3257–61. 24982329

[23] Klauder JVA. Case for diagnosis. [Xanthoma]. Arch Derm Syphilol. 1946;53:558. 20983429

[24] Zhi Y Duan Y Zhang H. Solitary juvenile xanthogranuloma with tibial involvement: a case report. Int J Clin Exp Pathol. 2015;8:164–70. 25755703 PMC4348839

[25] Agabegi SS Iorio TE Wilson JD Fischgrund JS. Juvenile xanthogranuloma in an adult lumbar spine: a case report. Spine (Phila Pa 1976). 2011;36:E69–73. 21192217 10.1097/BRS.0b013e318201b7f5

[26] Miranda P Lobato RD Ricoy JR Lagares A Ramos A. Xanthogranuloma of the choroid plexus of the third ventricle: case report and literature review. Neurocirugia (Astur). 2005;16:518–22. 16378134 10.1016/s1130-1473(05)70381-x

[27] Cao D Ma J Yang X Xiao J. Solitary juvenile xanthogranuloma in the upper cervical spine: case report and review of the literatures. Eur Spine J. 2008;17(Suppl 2):S318–323. 18228052 10.1007/s00586-008-0606-0 PMC2525901

[28] Jain A Mathur K Khatri S Kasana S Jain SK. Rare presentation of juvenile xanthogranuloma in the thoracic spine of an adult patient: case report and literature review. Acta Neurochir (Wien). 2011;153:1813–8. 21626171 10.1007/s00701-011-1057-7

